# Identification of Antioxidative Peptides Derived from *Arthrospira maxima* in the Biorefinery Process after Extraction of C-Phycocyanin and Lipids

**DOI:** 10.3390/md21030146

**Published:** 2023-02-24

**Authors:** Renao Bai, Trung T. Nguyen, Yali Zhou, Yong Diao, Wei Zhang

**Affiliations:** 1 School of Medicine, Huaqiao University, Quanzhou 362021, China; 2 Centre for Marine Bioproduct Development, College of Medicine and Public Health, Flinders University, Adelaide, SA 5042, Australia; 3 Aquaculture Laboratory, College of Science and Engineering, Flinders University, Adelaide, SA 5042, Australia; 4 Marine Bioproducts Cooperative Research Centre, Adelaide, SA 5042, Australia

**Keywords:** *Arthrospira maxima*, spent biomass, proteases, bioactive peptides, antioxidant activity, biorefinery

## Abstract

*Arthrospira maxima* has been identified as a sustainable source of rich proteins with diverse functionalities and bioactivities. After extracting C-phycocyanin (C-PC) and lipids in a biorefinery process, the spent biomass still contains a large proportion of proteins with potential for biopeptide production. In this study, the residue was digested using Papain, Alcalase, Trypsin, Protamex 1.6, and Alcalase 2.4 L at different time intervals. The resulting hydrolyzed product with the highest antioxidative activity, evaluated through their scavenging capability of hydroxyl radicals, superoxide anion, 2,2-diphenyl-1-picrylhydrazyl (DPPH), and 2,2′-azino-bis (3-ethylbenzothiazoline-6-sulfonic acid (ABTS), was selected for further fractionation and purification to isolate and identify biopeptides. Alcalase 2.4 L was found to produce the highest antioxidative hydrolysate product after four-hour hydrolysis. Fractionating this bioactive product using ultrafiltration obtained two fractions with different molecular weights (MW) and antioxidative activity. The low-molecular-weight fraction (LMWF) with MW <3 kDa had higher DPPH scavenging activity with the IC_50_ value of 2.97 ± 0.33 compared to 3.76 ± 0.15 mg/mL of the high-molecular-weight fraction (HMWF) with MW >3 kDa. Two stronger antioxidative fractions (F-A and F-B) with the respective significant lower IC_50_ values of 0.83 ± 0.22 and 1.52 ± 0.29 mg/mL were isolated from the LMWF using gel filtration with a Sephadex G-25 column. Based on LC-MS/MS analysis of the F-A, 230 peptides derived from 108 *A. maxima* proteins were determined. Notably, different antioxidative peptides possessing various bioactivities, including antioxidation, were detected with high predicted scores together with in silico analyses on their stability and toxicity. This study established knowledge and technology to further value-add to the spent *A. maxima* biomass by optimizing hydrolysis and fraction processes to produce antioxidative peptides with Alcalase 2.4 L after two products already produced in a biorefinery. These bioactive peptides have potential applications in food and nutraceutical products.

## 1. Introduction

Population growth, deforestation, and climate change have posed multiple severe challenges to the modern food industry [1,2]. The global population is projected to reach 9.5–10 billion by 2050, with the annual demand for dietary protein predicted to increase to 360–1250 million tons by then [3,4]. Conventionally, dietary proteins are mainly derived from animal sources such as pork, chicken, mutton, beef, eggs, and dairy products. Meeting such a high demand for proteins would require a 73% increase in meat production by 2050. A massive quantity of natural resources such as arable land, freshwater, and animal feed would be needed for such a dramatic increase [5,6]. Additionally, meat production is associated with global warming and greenhouse gas emission. It is responsible for around one-fifth of greenhouse gas emissions, making its development unsustainable [7]. Although conventional plant-based protein sources, such as soybeans, pulses, and oilseeds, account for 65% of the global protein requirement, expanding their production is limited due to the restricted availability of arable land [8]. Consequently, there is an urgent need to identify alternative protein sources to cater to a growing global population.

Recently, many protein-rich sources, such as insects, microbial proteins, seaweeds, and microalgae, have been used as alternative proteins in the food industry [9,10,11,12]. Amongst these, microalgae have been considered a promising source of edible proteins owing to their high protein content, well-balanced amino acids, and excellent ecological adaptation [10]. They can use sustainable carbon sources to produce biomass with high photosynthetic conversion efficiencies (6%) compared to terrestrial crop plants (3.5–4%) [2]. The arable land will be a restrictive factor for increasing agricultural productivity in some developing countries such as Southeast Asia, northern and central South America, and sub-Saharan Africa [13]. However, arable land is no longer a requirement for the cultivation of microalgae on a large scale [14]. All these advantages make microalgae ideal alternative protein sources for commercial exploitation.

As one of the most commercially valuable microalgae, *Arthrospira* spp. have the highest annual production, accounting for about 60% of the total microalgae production in the world [10]. A. maxima and A. platensis are the two main species of Arthrospira. C-PC is one of the most widely studied pigment-proteins derived from Arthrospira because it possesses various bioactivities and functionalities with a series of commercial applications [15,16]. Furthermore, protein-based bioproducts produced from Arthrospira, such as bioactive peptides, have also been extensively studied [17,18,19,20,21,22]. This is due to several interesting bioactivities, including antioxidant, antihypertensive, immunomodulatory, anticancer, and anti-obesity activities [18,19,20,21,23]. Among these, antioxidative activity has received more interest since it is associated with the development of many chronic diseases, such as congenital pulmonary fibrosis, chronic obstructive pulmonary disease, cancers, Alzheimer’s disease, arteriosclerosis, hypertension, cardiopathy, and type 2 diabetes [24,25,26]. The effects of oxidative stress can be mitigated by the introduction of antioxidants to scavenge reactive oxygen species (ROS) or reactive nitrogen species (RNS).

Most studies of *Arthrospira* bioactive peptides have only focused on bioproducts produced from proteins extracted from *Arthrospira* or *Arthrospira* raw biomass [17,18,20,27,28]. In our study, a biorefinery process of *Arthrospira* biomass has been developed, with C-PC and lipids extracted as the first two products [29]. After extracting C-PC and lipids, the protein content was 72.8% in the spent biomass [29]. However, most of the proteins in the spent biomass were water-insoluble, and it was hard to extract them by conventional methods. In this study, the spent biomass was further processed by enzymatic hydrolysis and fractionation into antioxidative peptides to extend multi-product recovery.

## 2. Results and Discussion

### 2.1. Screening Promising Enzymes for Producing Antioxidative Peptides from Spent Biomass of A. maxima after the Recovery of C-PC and Lipids

Although physical, chemical, and biochemical methods have been used to prepare bioactive peptides from different protein sources, enzymatic hydrolysis is preferable [30] due to its mild and environmentally friendly conditions [31,32]. In this study, five different enzymes, including Papain, Alcalase, Trypsin, Protamex 1.6, and Alcalase 2.4 L were investigated to screen their potential in producing antioxidative protein hydrolysates from spent biomass of Arthrospira maxima after the recovery of C-PC and lipids in a biorefinery process [29]. The optimal conditions of these enzymes used for hydrolyzing in this study are shown in Table 1.

**Figure 1:**
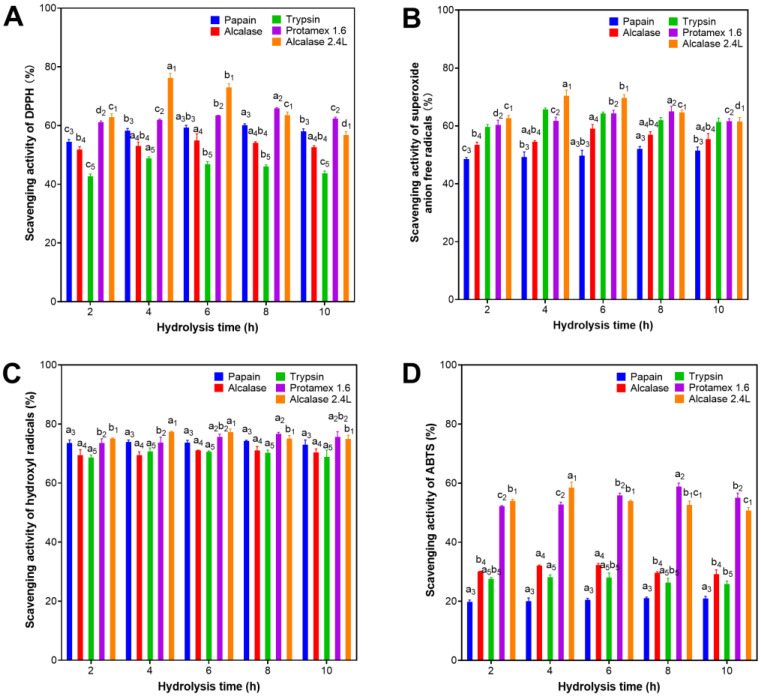
Antioxidant activity of protein hydrolysates of spent Arthrospira maxima biomass prepared with Papain, Alcalase, Trypsin, Protamex 1.6, and Alcalase 2.4 L for 2–10 h hydrolysis based on four different antioxidation assays: (**A**) scavenging activity of DPPH radicals, (**B**) scavenging activity of superoxide anion free radicals, (**C**) scavenging activity of hydroxyl radicals, and (**D**) total antioxidative capacity (T-AOC) (letters stand for a statistically significant difference with *p* < 0.05). The error bars represent the standard deviations of triplicates. The hydrolysis conditions are described in Table 1.

DPPH free radical scavenging activities of protein hydrolysates using five commercial proteases at different hydrolysis times are shown in Figure 1A. Protein hydrolysates generated from different enzymes have different antioxidative activity. Their DPPH scavenging activity were ranked in the following order: Alcalase 2.4 L > Protamex 1.6 > Papain > Alcalase > Trypsin. Alcalase 2.4 L was found to produce hydrolyzed products exerting the highest DPPH scavenging activity while Trypsin had the lowest. The DPPH scavenging activity of protein hydrolysates produced at different hydrolysis times were found to be statistically insignificant for most of the investigated enzymes except Alcalase 2.4 L (Figure 1A). The highest DPPH scavenging activity of 76.2% was observed at 4 h hydrolysis by Alcalase 2.4 L. The lowest DPPH scavenging activity of 42.7% was seen in the 2-h Trypsin hydrolysis. For other proteases, the best DPPH scavenging activity was achieved after 8 h for Papain (60.1%), 6 h for Alcalase (54.9%), 4 h for Trypsin (48.8%) and 8 h for Protamex 1.6 (65.9%).

The highest antioxidative activity around 70.4% of Alcalase 2.4 L hydrolyzed products with 4- and 6 h hydrolysis were also observed in the superoxide anion scavenging activity (Figure 1B). For the hydrolyzed products generated from other enzymes, however, their antioxidative activity had a different order: Alcalase 2.4 L > Protamex 1.6 > Trypsin > Alcalase > Papain. Trypsin was found to produce higher antioxidative hydrolysate quantified with the superoxide anion scavenging activity of 65.7% (4 h), while that of papain-hydrolyzed product was lower at 52.0% (8 h). Discrepancy in the antioxidative value between these two assays observed on the same hydrolysate could be explained by differences in scavenging mechanisms for different free radicals. 

Although antioxidative activity of protein hydrolysates produced from different enzymes and hydrolysis time showed differences on the DPPH and superoxide anion assays, their hydroxyl radical scavenging activity was found to be insignificantly different (Figure 1C). All had high activity values which ranged from 68.6% (2 h hydrolysis with trypsin) to 77.3% (4 h hydrolysis with Alcalase 2.4 L). The results are in contrast to their antioxidative activity measured with ABTS scavenging capacity (Figure 1D). Only hydrolyzed products derived from Alcalase 2.4 L and Protamex 1.6 had relatively high ABTS scavenging activity of around 58.8% after 4 and 8 h hydrolysis time, respectively. Meanwhile, the ABTS values of the hydrolysates derived from three other enzymes were much lower, around 30% for Alcalase and Trypsin, and 20% for Papain. Notably, antioxidative activity of the hydrolysates produced at different hydrolysis time was insignificant. In summary, Alcalase 2.4 L that hydrolyzed *A. maxima* proteins in the spent biomass had the highest antioxidative activities observed on all the four assays, particularly hydrolyzed products obtained at 4 h hydrolysis.

### 2.2. Antioxidative Activities of Peptide Fractions by 3-kDa Ultrafiltration

**Figure 2:**
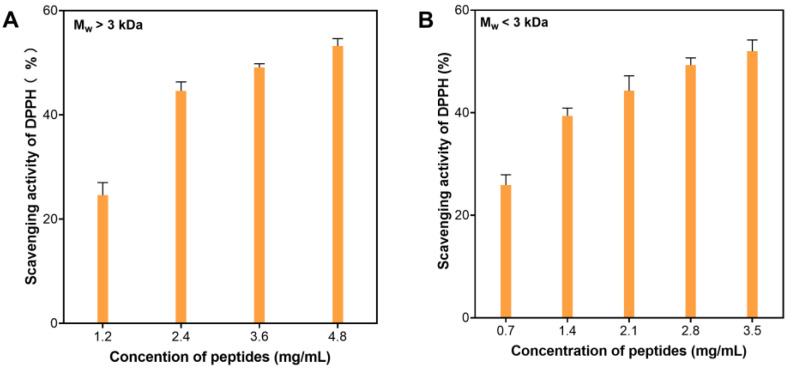
DPPH scavenging activity of peptide fractions from Alcalase 2.4 L hydrolysis of spent Arthrospira maxima biomass with different molecular weights at different concentrations: (**A**) MW > 3 kDa, (**B**) MW < 3 kDa.

As a result of the enzymatic hydrolysis screening experiments, protein hydrolysate generated from Alcalase 2.4 L hydrolysis was chosen as the optimal process for further investigation. The hydrolysis process was performed at 60 °C, pH 9.0, 4 h, and 2340U of Alcalase 2.4 L per gram of spent biomass sample. After hydrolysis and centrifugation, the supernatant was collected and precipitated with 80% ethanol (*v*/*v*). Supernatants collected from ethanol precipitation were evaporated in a fume hood to remove ethanol and filtered with 0.45 μm of sterile syringe filters. The filtrates were then subjected to centrifugal ultrafiltration to partition the peptides into two fractions with molecular weight (MW) <3 kDa (LMWF) and >3 kDa (HMWF). Antioxidative activity of these fractions was evaluated using DPPH scavenging. The LMWF had higher DPPH scavenging activity than that of the HMWF (IC_50_ = 2.97 ± 0.33 vs. 3.76 ± 0.15 mg/mL) (Figure 2). This result is consistent with reported observations that smaller peptides exhibited better antioxidative activities than larger peptides [30,33]. Therefore, the lower MW fraction was further purified using chromatography gel filtration.

### 2.3. Antioxidative Peptides Purified by Chromatography Gel Filtration

Ultrafiltration and gel filtration chromatography were used for the purification of protein hydrolysates prepared by Alcalase 2.4 L. The LMWF displayed better activity on scavenging DPPH radicals than the HMWF (Figure 2). Previous studies have proven that amino acid residues ranging from 5–11 showed good antioxidant activities [34,35]. The LMWF was further purified using the Sephadex G-25 chromatography to obtain two fractions named F-A and F-B. Over two hundred peptide sequences were identified in F-A. The antioxidant activity of these peptides was predicted using BIOPEP-UWM^TM^ database tools and five novel peptide sequences were chosen from these sequences. Herein, some novel antioxidative peptides were identified from non-phycobiliproteins such as C-PC, allophycocyanin, and phycoerythrin (Table 2 and Table 3). This is the first study to use the spent biomass of *A. maxima* to produce antioxidative peptides after C-PC and lipids were extracted. Most previous studies have used the raw biomass of *Arhthrospira* or water-soluble proteins extracted from *Arhthrospira* to prepare bioactive peptides. Almost the reported bioactive peptides were derived from the phycobiliproteins of *Arhthrospira* [35].

**Figure 3:**
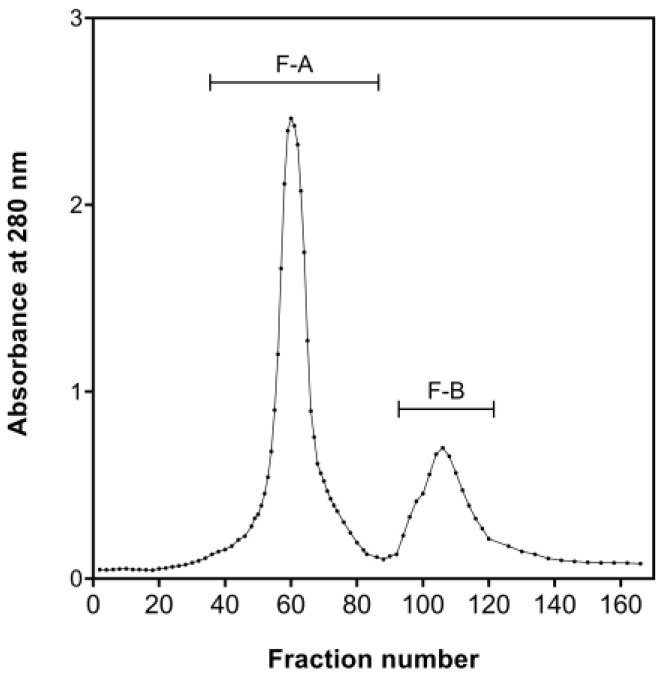
Chromatography gel filtration of the bioactive fraction with MW <3 kDa using Sephadex G-25.

The chromatography gel filtration was conducted using a Sephadex G-25 column. The eluted peptides were separated into two discrete peaks (peak maxima at fractions 60 and 110, respectively). The corresponding fractions were collected, pooled, and freeze-dried to obtain products defined as Fraction-A (F-A) and Fraction-B (F-B) (Figure 3).

**Figure 4:**
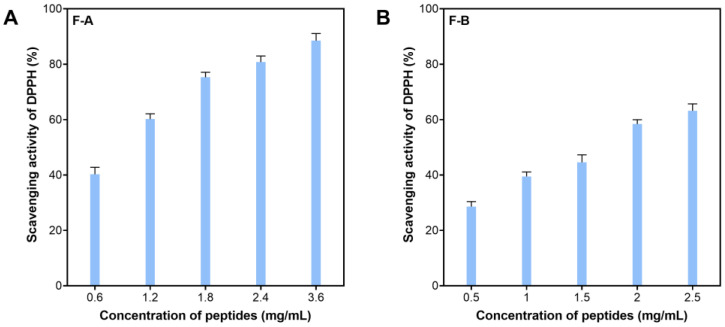
Scavenging activity of F-A (**A**) and F-B (**B**) on DPPH.

These two fractions had different antioxidative activity based on their IC_50_ values for DPPH scavenging activity. The F-A exhibited higher activity, indicated by the IC_50_ value (mg/mL) of 0.83 ± 0.22 compared to 1.52 ± 0.29 of the F-B (Figure 4). Higher antioxidation of the F-A could be explained by its high purity (a narrow peak). Additionally, its amino acid sequence and composition with a richness of hydrophobic amino acid could contribute to such high bioactivities. This was reported by several authors in different studies [30,36]. Therefore, the F-A was subjected to a further analysis with LC-MS/MS to identify the sequence of the active peptides.

### 2.4. Prediction of Bioactive Potential of Peptides Identified from LC-MS/MS Results

Numerous studies have found that a few key factors, such as molecular weight, amino acid compositions, and amino acid sequence, play critical roles in bioactivity [30,36]. Conventionally, in order to obtain a high purity of bioactive peptides, ion exchange chromatography and reverse-phase HPLC have been used to purify bioactive peptides. However, these processes are slow, cumbersome, and costly since they require a lot of laboratory work. A few models based on machine learning or deep learning have been used to predict the activities of peptides [37,38,39]. Herein, an online tool named Peptide Ranker (PepRank) was used to predict bioactive peptides resulting from LC-MS/MS [37]. It can help identifying the potential bioactive peptides from a wide range of peptide sequences in a short time.

Approximately 230 peptide sequences in the F-A were identified from the result of LC-MS/MS. These peptides were derived from 108 *Arthrospira* proteins. Their molecular masses ranged from 544.3 to 3194.5 Da, in which proportions of peptides with MW of 500–1000, 1000–2000, and >2000 Da accounted for 45.1, 48.3, and 6.6%, respectively. All peptide sequences were matched against an online in silico tool named Peptide Ranker (PepRank) (http://distilldeep.ucd.ie/PeptideRanker/ accessed on 20 December 2022) to identify peptides with bioactivity. For this in silico tool, selecting the general threshold value is crucial because it helps to reduce the false-positive rate. Thus, the threshold value of 0.5 was selected to screen bioactive peptides with higher potential. Twenty-two highly bioactive peptides were identified and selected with their predicted scores, sequences, molecular weight, and parent proteins, as illustrated in Table 2. The peptide KNAMPAFNGRL, derived from Cytochrome C6, was evaluated to have the highest bioactivity with a predicted score of 0.86. High bioactivity (score of 0.68–0.83) was also observed in peptides derived from light-harvesting proteins of chlorophyll a/b and some phycobilisome linkers. However, the predicted score of peptides generated from phycobilisome proteins and C-phycocyanin alpha subunit are relatively low, 0.52–0.54. The molecular weight of these bioactive peptides ranged from 893.0 Da (RAGGYTRL) to 1783.9 Da (KRPDFIAPGGNAAGQRE). The potential bioactivities of these peptides were also predicted by using the BIOPEP-UWM database. These potential bioactivities were presented in Table 2 and the detailed explanations of these bioactivities can be found in a publication by Minkiewicz et al. [40]. Herein, we focused on the peptides with the antioxidative activity, as other bioactivities are outside of the scope of this study.

**Table 2:** Bioactive potential of peptides identified from the F-A according to PEPRANK and BIOPEP-UWM database.

Peptide Sequence	PepRank	Molecular Weight (Da)	Potential Bioactivities	Protein Group
KNAMPAFNGRL	0.86	1218.4	ACE inhibitor, DPP IV inhibitor	Cytochrome C6
RALGFDFRR	0.83	1137.3	ACE inhibitor, Antioxidative, DPP IV inhibitor, DPP III inhibitor, Activating ubiquitin-mediated proteolysis	Chlorophyll a/b binding light-harvesting protein
KAPGFGDRR	0.78	1003.1	ACE inhibitor, DPP IV inhibitor, DPP III inhibitor, Antiamnestic, Antithrombotic, Regulating	60 kDa chaperonin
RHTPFFKG	0.77	989.1	ACE inhibitor, DPP IV inhibitor, Antioxidative	Elongation factor
RNPAIFRG	0.75	930.1	ACE inhibitor, DPP IV inhibitor	Carbohydrate-selective porin OprB
KFFYPNFQTRV	0.75	1446.7	ACE inhibitor, DPP IV inhibitor, Alpha-glucosidase inhibitor, Renin inhibitor, CaMPDE inhibitor	Phycobilisome linker polypeptide
RGQWTVGFNRM	0.71	1351.6	ACE inhibitor, DPP IV inhibitor, DPP III inhibitor, Renin inhibitor, Neuropeptide	Phycobilisome linker polypeptide
KFFYGNSQVRF	0.68	1392.6	ACE inhibitor, DPP IV inhibitor, DPP III inhibitor, CaMPDE inhibitor, Renin inhibitor, Immunomodulating	Phycobilisome linker polypeptide
KAGYLFPEIARR	0.67	1420.7	ACE inhibitor, DPP IV inhibitor, DPP III inhibitor, Renin inhibitor, Neuropeptide, Alpha-glucosidase inhibitor	LL-diaminopimelate aminotransferase
RDNVLRF	0.63	919	ACE inhibitor, DPP IV inhibitor, DPP III inhibitor, Renin inhibitor, Stimulating	Orange carotenoid protein
RIPPYRN	0.63	915.1	ACE inhibitor, DPP IV inhibitor, DPP III inhibitor, Alpha-amylase inhibitor, Anti-inflammatory, Alpha-glucosidase inhibitor	Polypeptide-transport-associated domain protein ShlB-type
RNLGAGSQFNLPRN	0.62	1543.7	ACE inhibitor, DPP IV inhibitor, DPP III inhibitor, Renin inhibitor	Extracellular solute-binding protein family 3
RSIPTLMIFKG	0.60	1262.6	ACE inhibitor, DPP IV inhibitor	Thioredoxin
RQMSLLLRR	0.60	1172.4	ACE inhibitor, DPP IV inhibitor, DPP III inhibitor, Renin inhibitor, Stimulating, Regulating, Antioxidative	Hypothetical protein
RLQLLARF	0.58	1016.2	ACE inhibitor, DPP IV inhibitor, DPP III inhibitor, Renin inhibitor, Stimulating, Antioxidative, Activating ubiquitin-mediated proteolysis	Methyltransferase type 11
RFGIISVRF	0.58	1094.3	ACE inhibitor, DPP IV inhibitor, DPP III inhibitor, Stimulating	Uncharacterized protein
KFVVGGPQGDSGLTGRK	0.58	1702.9	ACE inhibitor, DPP IV inhibitor, Regulating, Antiamnestic, Antithrombotic, Renin inhibitor, CaMPDE inhibitor	S-adenosylmethionine synthase
KVAINGFGRI	0.57	1074.3	ACE inhibitor, DPP IV inhibitor, DPP III inhibitor	Glyceraldehyde-3-phosphate dehydrogenase
KADSLISGAAQAVYNKF	0.54	1783.0	ACE inhibitor, DPP IV inhibitor, DPP III inhibitor, Stimulating, Regulating, Alpha-glucosidase inhibitor, CaMPDE inhibitor, Renin inhibitor, Hypotensive, Antioxidative	C-phycocyanin alpha subunit
KIGLFGGAGVGKT	0.54	1204.4	ACE inhibitor, DPP IV inhibitor, Regulating, Immunomodulating	ATP synthase subunit beta
RAGGYTRL	0.52	893.0	ACE inhibitor, DPP IV inhibitor, Activating ubiquitin-mediated proteolysis	Dihydroorotase
KRPDFIAPGGNAAGQRE	0.52	1783.9	ACE inhibitor, DPP IV inhibitor, Regulating, Antiamnestic, Antithrombotic, Immunomodulating, Hypotensive, Neuropeptide	Phycobilisome protein

A—Alanine, D—Aspartic acid, E—Glutamic Acid, F—Phenylalanine, G—Glycine, H—Histidine, I—Isoleucine, K—Lysine, L—Leucine, M—Methionine, N—Asparagine, P—Proline, Q—Glutamine, R—Arginine, S—Serine, T—Threonine, V—Valine, W—Tryptophan, Y—Tyrosine, ACE: Angiotensin-Converting Enzyme, DPP: Dipeptidyl Peptidase.

### 2.5. Antioxidative Peptides Identified Based on In Silico Analysis

BIOPEP-UWM^TM^ database tools were used to identify the antioxidative potential of the 22 bioactive peptides screened in the previous step. Sequences of these identified peptides were submitted to the BIOPEP as query sequences and “antioxidant activity” was selected as a bioactivity of interest. Table 3 shows the antioxidant potential prediction of these biopeptides. According to the BIOPEP-UWM^TM^, only five antioxidative peptides were identified. All the biopeptides were detected to contain 2–3 amino acid antioxidative fragments, except for KADSLISGAAQAVYNKF. This biopeptide could produce two bioactive sequences (GAA and VY). Hydrophobic amino acids (HAAs) were found in all the active fragments. HAAs such as leucine (L), glycine (G), alanine (A), phenylalanine (F), valine (V), threonine (T) and tyrosine (Y) are considered the key attributes to the antioxidative property of these biopeptides [41,42,43]. For example, phenylalanine can act as a hydrogen donor to scavenge free radicals [44]. Another reason is that the presence of hydrophobic amino acids can increase the lipid solubility of these biopeptides to promote their interactions with different radical species [44,45]. In addition, other amino acid residues in the sequence may also play an important role in the antioxidative properties of peptides [46,47]. Therefore, these characters are important to identify novel antioxidant peptides.

**Table 3:** Antioxidative peptides identified from 22 screened biopeptides based on their in silico analysis.

Peptide Sequences	Activity	Bioactive Sequences	Fragmentation Locations
RALGFDFRR	Antioxidative	LGF	(3-5)
RHTPFFKG	Antioxidative	RHT	(1-3)
RQMSLLLRR	Antioxidative	LLR	(6-8)
RLQLLARF	Antioxidative	LQL	(2-4)
KADSLISGAAQAVYNKF	Antioxidative	GAA	(9-11)
KADSLISGAAQAVYNKF	Antioxidative	VY	(14-15)

A—Alanine, D—Aspartic acid, F—Phenylalanine, G—Glycine, H—Histidine, I—Isoleucine, K—Lysine, L—Leucine, M—Methionine, N—Asparagine, P—Proline, Q—Glutamine, R—Arginine, S—Serine, T—Threonine, V—Valine, Y—Tyrosine.

### 2.6. In Silico Simulated Gastrointestinal Digestion of the Antioxidative Peptides

The bioavailability and bioaccessibility of peptides in vivo are vital to their applications because biopeptides may be degraded and lose their bioactivities during gastrointestinal digestion. Therefore, it is necessary to study the in vitro stability of these biopeptides against gastrointestinal digestion by mimicking protein degradation in the stomach and small intestine [40]. In this regard, the “BIOPEP Enzyme (s) Action” program was used to simulate the digestion process of the five selected antioxidative peptides. Three digestive enzymes, including pepsin (EC 3.4.23.1), trypsin (EC 3.4.21.4), and chymotrypsin (EC 3.4.21.1), were used to mimic the process of in silico proteolysis. Table 4 represents the bioactive potential of the five antioxidative peptides after being hydrolysed by gastrointestinal proteases.

After the in silico digestion, none of the peptides showed antioxidative potential. This means that these biopeptides are unstable and they lose their antioxidative activity after gastrointestinal digestion. This may be related to the lack of information in databases related to the antioxidant bioactivities of peptides [48]. Another study also reported the same results, and a few antioxidant peptides lost their bioactivity after digestion by gastrointestinal proteases [49]. According to the predictions, ACE and dipeptidyl peptidase IV inhibitory peptides were found from their original sequences. In addition, these bioactive peptides were all dipeptides. Other quantitative parameters, such as theoretical degree of hydrolysis (DH%), the frequency of the release of fragments with a given activity by selected enzymes (A_E_), and the relative frequency of the release of fragments with a given activity by selected enzymes (W), were calculated (Table 5). The DH values ranged from 18.75 to 57.14. RHTPFFKG had the highest efficiency (57.14%) in releasing the bioactive peptides and KADSLISGAAQAVYNKF had the lowest (18.75%). A_E_ and W values can be used to describe the potential bioactivity of *Arthrospira*-derived peptides. The higher A_E_ values represent a higher number of peptides with specific activity. RALGFDFRR showed the highest A_E_ values (0.33) in all peptide sequences, implying that more bioactive peptides can be derived from this peptide (Table 5) [49], while W values are related to the number of specific catalytic sites in each enzyme [50]. Most of the bioactive fragments with ACE inhibitory activities contained phenylalanine (F), glycine (G), leucine (L), and arginine (R) after in silico simulated digestion (Table 5). These characteristics have been reported in other studies [50,51]. These findings can provide more information in understanding the relationship between the amino acid composition of peptides and their stability in vivo.

**Table 4:** Remaining bioactive properties after in silico simulated gastrointestinal digestion of the antioxidative peptides.

Peptides	Results of Enzyme Action	Locations of Released Peptides	Active Fragment Sequence	Location	Bioactivities of Identified Peptide
RALGFDFRR	RAL-GF-DF-RR	(1-3) (4-5) (6-7) (8-9)	GF RR DF	(4-5) (8-9) (6-7)	ACE inhibitor, Dipeptidyl peptidase IV inhibitor
RHTPFFKG	RH-TPF-F-K-G	(1-2) (3-5) (6-6) (7-7) (8-8)	RH	(1-2)	Dipeptidyl peptidase IV inhibitor
RQMSLLLRR	RQM-SL-L-L-RR	(1-3) (4-5) (6-6) (7-7) (8-9)	RR SL	(8-9) (4-5)	ACE inhibitor, Dipeptidyl peptidase IV inhibitor
RLQLLARF	RL-QL-L-ARF	(1-2) (3-4) (5-5) (6-8)	RL QL	(1-2) (3-4)	ACE inhibitor, Dipeptidyl peptidase IV inhibitor
KADSLISGAAQAVYNKF	KADSL- ISGAAQAVY-N-KF	(1-5) (6-14) (15-15) (16-17)	KF	(16-17)	ACE inhibitor Dipeptidyl peptidase IV inhibitor

A—Alanine, D—Aspartic acid, G—Glycine, H—Histidine, I—Isoleucine, K—Lysine, L—Leucine, M—Methionine, N—Asparagine, P—Proline, Q—Glutamine, R—Arginine, S—Serine, T—Threonine, V—Valine, Y—Tyrosine, ACE: Angiotensin-Converting Enzyme.

**Table 5:** In silico hydrolysis performance and physicochemical characteristics of bioactive peptides remaining after simulated in silico digestion.

Peptide	Active Fragment Sequence	Location	DH (%)	A_E_	W
RALGFDFRR	GF RR DF	(4-5) (8-9) (6-7)	37.50	0.33	0.50
RHTPFFKG	RH	(1-2)	57.14	0.13	0.17
RQMSLLLRR	RR SL	(8-9) (4-5)	50.00	0.11	0.50
RLQLLARF	RL QL	(1-2) (3-4)	42.86	0.13	0.17
KADSLISGAAQAVYNKF	KF	(16-17)	18.75	0.06	0.10

A—Alanine, D—Aspartic acid, F—Phenylalanine, G—Glycine, H—Histidine, I—Isoleucine, K—Lysine, L—Leucine, M—Methionine, N—Asparagine, P—Proline, Q—Glutamine, R—Arginine, S—Serine, T—Threonine, V—Valine, Y—Tyrosine.

### 2.7. Prediction of Toxicity and Physicochemical Properties of Released Bioactive Peptide Fractions after In Silico Digestion

Although peptides derived in silico proteolysis did not show antioxidative activity as we expected, it was necessary to analyze the safety of the resultant peptides for their further use in the food and pharmaceutical industries. The toxicity and other physicochemical properties, such as hydrophobicity, hydrophilicity, charge, isoelectric point (pI), and molecular weight, of the released dipeptides were predicted by the software ToxinPred. The results indicated that the eight dipeptides were safe and their molecular weight ranged from 218.27 to 330.40 Da. Three (GF, SL, and QL) out of eight identified ACE inhibitory dipeptides had a pI of 5.88 and 0 net charge (Table 6).

RR, RH, RL, and KF had a positive charge varying from 1.00 to 2.00 with pI ranging from 9.11 to 12.01. DF was the only dipeptide with negative charge (−1) and the lowest pI of 3.8. Hydrophobicity values of identified bioactive dipeptides varied from −1.76 to 0.39, while hydrophilicity values ranged from −1.25 to 3.00. These identified bioactive dipeptides can be applied in both hydrophilic and hydrophobic systems.

**Table 6:** Physicochemical properties and toxicity of bioactive fragments released after the simulated in silico digestion.

Peptide	Prediction	Hydrophobicity	Hydrophilicity	Charge	pI	Molecular Weight (Da)
GF	Non-Toxic	0.39	−1.25	0.00	5.88	222.26
RR	Non-Toxic	−1.76	3.00	2.00	12.01	330.40
DF	Non-Toxic	−0.05	0.25	−1.00	3.8	280.29
RH	Non-Toxic	−1.08	1.25	1.50	10.11	311.36
SL	Non-Toxic	0.14	−0.75	0.00	5.88	218.27
RL	Non-Toxic	−0.61	0.60	1.00	10.11	287.38
QL	Non-Toxic	−0.08	−0.80	0.00	5.88	259.33
KF	Non-Toxic	−0.25	0.25	1.00	9.11	293.38

D—Aspartic acid, F—Phenylalanine, G—Glycine, H—Histidine, K—Lysine, L—Leucine, Q—Glutamine, R—Arginine, S—Serine.

## 3. Methods

### 3.1. Materials

Alcalase 2.4 L and Protamex 1.6 were provided by Novozymes (Beijing, China). Papain and Alcalase were obtained from Pangbo Bioengineering Co., Ltd. (Nanning, China). Trypsin was purchased from Sunson Biotechnology Co., Ltd. (Cangzhou, China). Sodium carbonate, trichloroacetic acid, methyl aldehyde, glacial acetic acid, sodium hydroxide, and absolute ethyl alcohol were purchased from Sinopharm (Shanghai, China). 2,2-Diphenyl-1-picrylhydrazyl (DPPH) and Folin & Ciocalteu’s phenol reagent were obtained from Sigma-Aldrich (Shanghai, China). Kits for measuring scavenging activities of hydroxyl free radicals, superoxide anion free radicals and 2,2′-Azino-bis (3-ethylbenzothiazoline-6-sulfonic acid) diammonium salt (ABTS) were provided by Nanjing Jiancheng Bioengineering Institute (Nanjing, China). The BCA protein assay kit (P0012) was bought from Beyotime Biotechnology (Shanghai, China).

### 3.2. C-PC and Lipids Extracted to Generate the Spent Biomass

Dried biomass was mixed with Milli-Q water at a ratio of 20 mL/g and then sonicated at an amplitude of 80% for 16 min by a sonicator at 750 W and 20 kHz with a probe diameter of 25 mm (Vibro-Cell, SONICS & Materials, Newtown, CT, USA). The disrupted cell slurry was adjusted to pH 4.8 using acetic acid 0.5 M solution to facilitate a recovery of high purity C-PC based on an unpublished study by our group [29]. The pH-adjusted slurry was centrifuged at 2500× *g* for 30 min to obtain the supernatant for C-PC recovery, while the insoluble precipitates, debris, and fragments were collected and frozen in the freezer at −80 °C before being freeze-dried at −85 °C, 13 mTorr for around 48 h to obtain the pellet (the total yield of 69.2%) which was used for further extraction of pigments using acetone/ethanol/water (AEW) at a ratio of 2:3:5 (*v/v/v*) in a 250 mL flask. The flask was shaken under dark conditions at an ambient temperature (20–22 °C) at 150 rpm for 24 h on a shaker (Ratek, Medium reciprocating shaker, RM2, Boronia, Australia). Then, the mixture was filtered through a 0.45 μm membrane (Millipore PTFE, FGLP04600, 47 mm) under the vacuum condition. The cake was collected for the second extraction with the same procedures. Finally, the cake was collected and air-dried in a fume hood before it was ground with a mortar and pestle into fine particles at a yield of 53.6%, defined as the spent biomass, and stored in the freezer for further use.

### 3.3. Enzymatic Hydrolysis

The hydrolysis conditions of the commercial proteases were optimized before further processing. Briefly, 0.25 g of spent biomass was mixed with 10 mL of distilled water to obtain the homogenized mixture which was adjusted to the optimum pH and temperature values according to the manufacturer’s recommendations for each enzyme (Table 1). After reaching the targeted pH and temperatures, proteases were added to commence hydrolysis. Samples were mixed every 20 min over a total hydrolysis time of 2–10 h. After hydrolysis, the samples were heated to 85 °C for 15 min to inactivate proteases and then cooled to room temperature (20–22 °C) before they were centrifuged at 4 °C, 8000× *g* for 30 min. The collected supernatants were precipitated with four volumes of absolute ethanol, followed by centrifugation at 8000× *g* for 30 min at 4 °C. The supernatants were collected and evaporated in the fume hood to remove ethanol. The aqueous residue was filtered using sterile syringe filters with a 0.45 μm membrane (SLHPM33RS, Millipore, Darmstadt, Germany).

### 3.4. Antioxidation Assays

#### 3.4.1. Scavenging Activities of DPPH

The DPPH radical scavenging assay was performed following a modified protocol described by Tejano et al. [52]. Briefly, for the sample group, 100 μL DPPH 0.2 mM solution, freshly prepared by dissolving in ethanol 95%, were mixed with 100 μL of protein hydrolysates (4 mg/mL) in a 96-well plate. The plate and its contents were incubated at room temperature (22–25 °C) in the dark for 30 min before the absorbance was measured with a plate reader ReadMax 1900 (Flash, Shanghai, China) at 517 nm and recorded as A_s_. For the blank groups, 100 μL of distilled water was mixed with 100 μL of DPPH 0.2 mM solution and its absorbance was recorded as A_b_. For the control groups, 100 μL of protein hydrolysates was mixed with 100 μL of 95% ethanol and its absorbance was recorded as A_c_. The scavenging activity of protein hydrolysate on DPPH radicals can be calculated based on Equation (1):

(1) $$R=\left(1-\frac{A_{s}-A_{c}}{A_{b}}\right)$$

#### 3.4.2. Scavenging Activities of Hydroxyl Free Radicals

Scavenging activities of hydroxyl free radicals were measured as described by previous study [53]. A commercial kit (Nanjing Jiancheng Bioengineering Institute; Product code A018-1-1, Colorimetric method) was used according to the manufacturer’s protocol. Briefly, for the sample groups, 200 μL of protein hydrolysates (4 mg/mL) were mixed with 200 μL reagent-2, and 400 μL reagent-3 in a 10 mL colorimetric tube. All samples were incubated at 37 °C for 1 min. Then, 2 mL of color developing agent was added into each colorimetric tube and mixed well. All samples were incubated at room temperature (22–25 °C) in the dark for 20 min. Milli-Q water was used in the control group. Absorbances of samples (A_sample_) and the control (A_control_) was measured at a wavelength of 550 nm using a visible spectrophotometer (Shanghai Metash Instrument Co., Ltd., Shanghai, China). The scavenging activity of protein hydrolysate based on hydroxyl free radicals was calculated by Equation (2) as follows:

(2) $$R=\left(\frac{A_{\mathrm{control}}-A_{\mathrm{sample}}}{A_{\mathrm{control}}}\right)$$

#### 3.4.3. Scavenging Activities of Superoxide Anion Free Radicals

Scavenging activities of superoxide anion free radicals were quantified according to the previous report [54]. A superoxide anion assay kit (Nanjing Jiancheng Bioengineering Institute; Product code A052-1-1, Colorimetric method) was used based on the manufacturer’s protocol. Briefly, 50 μL of protein hydrolysates (4 mg/mL) were mixed with 1.0 mL reagent-1, 100 μL reagent-2, 100 μL reagent-3, and 100 μL reagent-4 in a 10 mL colorimetric tube. Milli-Q water and 0.15 mg/mL of vitamin C were used in the control group and standard, respectively. After vortex mixing for a few seconds, all colorimetric tubes were incubated at 37 °C for 40 min. Then, 2.0 mL of color-developing agent were added into each colorimetric tube and mixed well. All samples were incubated at room temperature (22–25 °C) in the dark for 10 min. Absorbances of the sample and control groups were recorded as A_sample_ and A_control_, respectively, at 550 nm using a visible spectrophotometer (Shanghai Metash Instrument Co., Ltd., Shanghai, China) and each sample in triplicate. The scavenging activity of protein hydrolysate based on superoxide anion free radicals was calculated as per Equation (2).

#### 3.4.4. Measurement of the Total Antioxidative Capacity

The total antioxidative capacity was measured as described in the previous report [55]. Total antioxidant capacity was conducted according to the manufacturer’s protocol (Nanjing Jiancheng Bioengineering Institute; Product code A015-2-1, ABTS method). Briefly, 10 μL of protein hydrolysates (4 mg/mL) were mixed with 20 μL reagent-4 and 170 μL ABTS working solution in a 96-well plate. The plate with its content was incubated at room temperature (22–25 °C) in the dark for 6 min before the absorbance was measured at a wavelength of 405 nm recorded as A_1_ using a plate reader (ReadMax 1900Flash, Shanghai, China). The Trolox solution (1 mM) was used as the standard with its absorbance recorded as A_0_. All measurements were performed in triplicates. The scavenging activity of protein hydrolysate based on ABTS radicals was computed as described in Equation (3).

(3) $$R=\left(\frac{A_{0}-A_{1}}{A_{0}}\right)$$

### 3.5. Ultrafiltration to Fractionate Peptides into Two Fractions with Different Molecular Weights and Antioxidative Activity

A centrifugal filter unit (Amicon^®^ Ultra-15, 3 kDa MWCO, 15 mL sample volume) was used to partition the ethanol-precipitated peptides to molecular weight species of >3 kDa and <3 kDa. Samples were centrifuged at 5000× *g* for 60 min at 4 °C. The two fractions were stored at −20 °C for further studies. The fractions with high antioxidative activity were subjected to gel filtration chromatography.

### 3.6. Chromatographical Gel Filtration to Purify Bioactive Peptides

The fractions with higher antioxidative activity obtained from ultrafiltration were purified by gel filtration chromatography. A 2.5 cm internal diameter × 50 cm (246 mL) Sephadex G-25 (9041-35-4, Sigma-Aldrich, Shanghai, China) column was used for purification. The column was pre-equilibrated with distilled water for 5–10 h prior to loading. Samples were loaded at 4% of column volume (10 mL) and the peptides were eluted at a rate of 1 mL/min with distilled water. Peptide elution was monitored by absorbance at 280 nm and 3 mL aliquots were taken over the duration of each run. Fractions corresponding to each discrete peak were collected and pooled for freeze-drying. Then, the freeze-dried products were stored at −20 °C for further analyses.

### 3.7. LC-MS/MS Analysis of Peptides

The highest antioxidative fractions obtained from the gel filtration chromatography were subjected to amino acid sequencing. Samples were shipped to Applied Shanghai Protein Technology Co., Ltd. (Shanghai, China) for LC-MS/MS analysis. Protein digestion was performed based on the filter-aided sample preparation procedure described by Wisniewski et al. [56]. The analysis was performed on a Q Executive Mass Spectrometer coupled to Easy nLC (Thermo Fisher Scientific, Waltham, MA, USA). MS/MS spectra were cross-referenced using MASCOT engine (Matrix Science, London, UK; version 2.2) against a nonredundant International Protein Index Arabidopsis sequence database v3.85 (released in September 2011; 39679 sequences) from the European Bioinformatics Institute (http://www.ebi.ac.uk/ (accessed on 15 August 2021)). For protein identification, the following options were used. Peptide mass tolerance = 20 ppm, MS/MS tolerance = 0.1 Da, Enzyme = Trypsin, Missed cleavage = 2, Fixed modification: Carbamidomethyl (C), Variable modification: Oxidation(M).

### 3.8. Prediction of Bioactive Potential of Identified Peptides from the LC-MS/MS Results

An online tool named PepRank (http://distilldeep.ucd.ie/PeptideRanker/ (accessed on 20 December 2022))was used to predict identified peptides from the LC-MS/MS according to their bioactive potential. According to the guidelines of this tool, peptide sequences were pasted into the field and each peptide on a separate line. In the next step, the predict button was clicked to start the prediction. After completing the analysis, the results can be copied to an Excel file for further processing. The threshold of 0.5 was selected to screen peptides with potential bioactivities. Selected peptides were analyzed for their antioxidative and other bioactivities using the BIOPEP-UWM^TM^ database (https://biochemia.uwm.edu.pl/biopep-uwm/ (accessed on 20 December 2022)) of bioactive peptides. The identified sequences with antioxidant activities were subjected to in silico hydrolysis using the enzyme action feature of BIOPEP-UWM^TM^ by employing pepsin (EC 3.4.23.1), trypsin (EC 3.4.21.4), and chymotrypsin A (EC 3.4.21.1) as representative digestive enzymes as described in [57].

### 3.9. Toxicity and Physicochemical Properties of Bioactive Peptides Released after In Silico Proteolysis

The potential toxicity, hydrophobicity, hydrophilicity, charge, isoelectric point (pI), and molecular weight of the peptides released after simulated digestive proteolysis were predicted using the ToxinPred (https://webs.iiitd.edu.in/raghava/toxinpred/index.html (accessed on 20 December 2022) web-based application according to the method described by Gupta [58].

### 3.10. Statistical Analysis

Statistical analyses were performed using IBM SPSS Statistics (IBM, Chicago, NY, USA), and two-way ANOVA followed by Bonferroni post hoc tests were appropriate. Data were expressed as mean ± standard error of the mean of triplicates. Value of *p* < 0.05 represents statistical significance. The IC_50_ value was calculated by Graphpad Prism 8 (Graphpad Software Inc., San Diego, CA, USA).

## 4. Conclusions

The spent biomass of *A. maxima* generated from the biorefinery process after extraction of C-PC and lipids is a great protein source to produce antioxidative and bioactive peptides. Alcalase 2.4 L was found to produce protein hydrolysates with the highest antioxidation activities, particularly the product of four-hour hydrolysis. Higher antioxidative activity fractions with the IC_50_ values of 2.97 ± 0.33, and then 0.83 ± 0.22 mg/mL, were obtained after centrifugal ultrafiltration and chromatographical gel filtration. Approximately 230 peptides derived from 108 *A. maxima* parent proteins were identified in the bioactive fraction. Notably, twenty-two peptides with different bioactivities, such as antihypertension, anti-diabetes, anticancer, and antioxidation were detected. These biopeptides had high scores of bioactivity. In particularly, biopeptides derived from proteins of elongation and cytochrome C6 could gain a bioactivity score of 0.77–0.84 for different bioactivities, including antioxidation. Five specific antioxidative peptides could be produced from these bioactive peptides. Although these antioxidative peptides are nontoxic, they are unstable and lose their antioxidative activity under gastrointestinal conditions. However, gastrointestinal hydrolysis of these biopeptides could produce several new highly bioactive fragments with antihypertensive and antidiabetic activities which are promising for healthy food, functional beverages, and nutraceuticals. This finding suggests that the spent biomass generated from the C-PC and lipid extraction, instead of being utilized for producing antioxidative peptides, should be designed for the production of the bioactive fractions with a wider range of bioactivities for multiple value-added applications.

## Figures and Tables

**Table 1:** Enzymes and their optimal conditions used for protein hydrolysis of spent *Arthrospira maxima* biomass.

Protease	Temperature/°C	pH	Dosage of Enzyme (U/g-Sample)	Unit of Activity (U/g-Enzyme)
Papain	45	7.5	6600	1.1 × 10^5^
Alcalase 2.4 L	60	9.0	2340	3.9 × 10^4^
Protamex 1.6	55	7.5	9000	1.5 × 10^5^
Trypsin	50	9.0	10,200	1.7 × 10^5^
Alcalase	55	8.5	14,400	2.4 × 10^5^

## Data Availability

Data are contained within the article.
